# Expanded View of NMR Spin–Lattice Relaxation in Fluorine-Containing Ionic Liquids

**DOI:** 10.1021/acs.jpclett.5c01665

**Published:** 2025-07-21

**Authors:** Giselle de Araujo Lima e Souza, Elizabeth Brandwein, Emilia Pelegano-Titmuss, Phillip Stallworth, Yong Zhang, Pedro José Oliveira Sebastião, Steven Greenbaum

**Affiliations:** † Department of Physics, 5924 Hunter College, CUNY, New York, New York 10065, United States; ¶ Department of Chemistry, 14780 New York City College of Technology, New York, New York 11201, United States; § Department of Chemical and Biomolecular Engineering, 6111 University of Notre Dame, Notre Dame, Indiana 46556, United States; ‡ CeFEMA and Department of Physics, Instituto Superior Técnico, Universidade de Lisboa, Lisboa 1049-001, Portugal

## Abstract

Fluorine-containing anions are widely used in ionic liquids due to their unique physicochemical properties. However, the local dynamics of both cations and anions and their associated relaxation mechanisms remain incompletely understood. Here, we present a ^1^H and ^19^F spin–lattice relaxation rate (*R*_1_) study as a function of frequency over a broad frequency range from 30 kHz to 800 MHz for ionic liquids containing BF_4_^–^, PF_6_^–^, TFSI^–^, and FSI^–^ anions and EMIM^+^ cation. By combining experimental *R*_1_^H^ and *R*_1_^F^ NMR dispersion (NMRD) profiles with relaxation models for both dipolar spin interactions and chemical shift anisotropy (CSA) contributions, we demonstrate that CSA is needed to accurately describe the *R*_1_^F^ relaxation behavior above ∼300 MHz, the extent of which depends on the anion structure. These findings challenge the long-standing assumption that dipolar contribution is the main source of ^19^F relaxation in these systems and highlight the importance of including CSA to accurately interpret ^19^F relaxation in ionic liquids, particularly at high frequencies. This work provides new insights into the molecular dynamics of fluorine-containing species.

## Graphical abstract

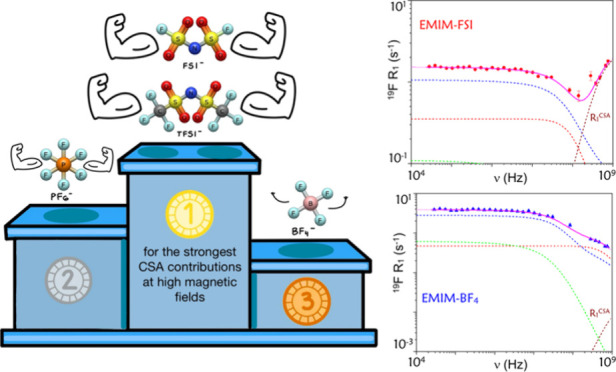

Ionic liquids (ILs) are salts that remain liquid at temperatures below 100 °C and are characterized by a dynamic ionic lattice that lacks long-range order, which can be either positional as found in crystals or orientational as found in liquid-crystals.^1^ However, they exhibit significant local structuring due to strong intermolecular interactions.^2–5^ This complex and thermally fluctuating environment governs the molecular dynamics, influencing physical properties at different length scales, including macroscopic properties such as viscosity, ionic conductivity, and transport behavior. A molecular-level understanding of these dynamics is essential for optimizing ILs for applications ranging from electrochemical devices to separation technologies. Nuclear magnetic resonance (NMR) relaxometry provides a powerful approach to probe molecular motions by measuring the spin–lattice relaxation rate (*R*_1_), where *R*_1_ is the reciprocal of the spin–lattice relaxation time, *T*_1_, as a function of the Larmor frequency (ω_L_= 2πν), a method known as NMR dispersion (NMRD).^6^ Within this framework, only a combination of conventional and fast field cycling (FFC) NMR techniques makes it possible to measure *R*_1_ over several orders of magnitude in frequency, from tens of kilohertz to hundreds of megahertz, offering the possibility to obtain detailed access to dynamical processes in the range from microsecond to nanosecond.^7^

In ionic liquids, the relaxation of spin ^1^/_2_ nuclei such as ^1^H and ^19^F is driven by time-dependent fluctuations in local magnetic fields caused by molecular motions.^8–12^ These fluctuations arise primarily from both intra- and intermolecular dipolar interactions and have traditionally been considered to be the dominant source of spin–lattice relaxation. The assumption that these fluctuations have autocorrelation functions that typically decay exponentially with time, characterized by specific correlation times, has formed the basis for modeling ^1^H and ^19^F spin–lattice relaxation. Thus, the relaxation spectral densities can be expressed in terms of the Lorentzian functions. This is the case of the very often used Bloembergen–Purcell–Pound (BPP) model^13–17^ or generalizations of this model that incorporate a distribution of correlation times, such as the Cole–Davidson model.^6,18,19^ While this framework is widely considered in the context of small and symmetric molecules, it relies on the assumption of an isotropic reorientation and a single averaged correlation time. This simplification may not be suitable to describe rotations/reorientations of elongated or asymmetric molecules, where molecular reorientations can involve distinct dynamics along different molecular axes.^20^ In such systems, the use of a single correlation time to describe rotational motion can result in an oversimplified description of the rotation/reorientation relaxation mechanism, particularly when analyzing NMR dispersion data over a very broad range of Larmor frequencies.

Additionally, translational molecular motions also contribute to modulating the dipolar spin interactions and are frequently described using the force-free hard-sphere (FFHS) model, which assumes that molecules behave as rigid spheres with nuclear spins located at their centers.^21^ Despite its simplicity, the FFHS approach has been extensively applied with success as it captures the continuous nature of ionic motion and its impact on relaxation rates. This framework is grounded in the model developed by Hwang and Freed,^22^ which relates self-diffusion to the time-dependent displacement of spin-bearing particles and defines the characteristic correlation time as τ = *d* ^2^/2*D*, where *d* is the internuclear distance and *D* is the self-diffusion coefficient. However, in ionic liquids, the absence of long-range order and the formation of transient ion pairs challenge the assumption of a constant internuclear geometry, potentially limiting the applicability of this approach. Alternatively, the model introduced by Torrey^23^ and modified by Harmon and Muller^24^ can describe both “jump” diffusion processes and continuous diffusion as a limiting case, using the mean square displacement ⟨*r* ^2^⟩ to define the characteristic correlation time, given by τ = ⟨*r* ^2^⟩/6*D*. This approach offers a description of diffusion-driven dipolar relaxation in systems where ionic motion is not strictly continuous and has also been adopted to describe the translational dynamics in ionic liquids.^25,26^

One general feature of the relaxation models associated with the dipolar spin interactions is the decrease of the relaxation rate with increasing frequency when the Larmor frequencies become larger than the characteristic correlation times of the molecular motions. However, at higher magnetic fields, the field dependence of ^19^F relaxation rates in ionic liquids introduces additional complexity. It is known that the chemical shift anisotropy (CSA) can be an effective mechanism, particularly in the case of ^19^F relaxation in small molecules,^27^ because the CSA spin–lattice relaxation contribution is proportional to the square of the magnetic field strength.^28^ Despite this theoretical foundation,^29^ CSA has often been overlooked in studies of ionic liquids, partly due to the assumption that rapid molecular motion averages out the anisotropic part of the chemical shift interaction.^30–34^ However, while this averaging suppresses the spectral manifestation of CSA, the fluctuations of the anisotropic interaction do not average out and might contribute significantly to spin–lattice relaxation.^27,35^ As the interest in understanding the full relaxation behavior of ^19^F nuclei in ILs is continuously increasing, particularly at high magnetic fields, there is a need to revisit the role of the CSA and reconsider the limitations of describing the relaxations on the basis of models that account only for dipolar spin interactions.

To address this gap and provide a clearer understanding of local dynamics probed by ^19^F relaxation, we investigated a series of ionic liquids composed of the 1-ethyl-3-methylimidazolium (EMIM^+^) cation and four fluorine-containing anions with distinct size and symmetry: bis­(trifluoromethanesulfonyl)­imide (TFSI^–^), bis­(fluorosulfonyl)­imide (FSI^–^), hexafluorophosphate (PF_6_^–^), and tetrafluoroborate (BF_4_^–^) (Figure 1). The ionic liquids were obtained from IoLiTec; all sample preparation and handling were performed under an argon atmosphere, and the samples were flame-sealed in NMR tubes to avoid moisture absorption and air exposure. ^1^H and ^19^F nuclei were used to selectively probe the dynamics of the cations and anions, respectively, allowing for a direct comparison of their individual motional behavior. Longitudinal relaxation times (*T*_1_) were measured by using both FFC and conventional fixed-field NMR techniques, and relaxation rates (*R*_1_ = 1/*T*_1_) were calculated accordingly. To reduce the number of unknown parameters in the relaxation model fitting, self-diffusion coefficients were measured independently by Pulsed Field Gradient (PFG) NMR, and the intra- and intermolecular distances were estimated from molecular dynamics (MD) simulations. This approach helped to constrain the physically meaningful range of distances used in the fitting process. Further experimental and MD simulation details are provided in the Supporting Information.

**Figure 1:**
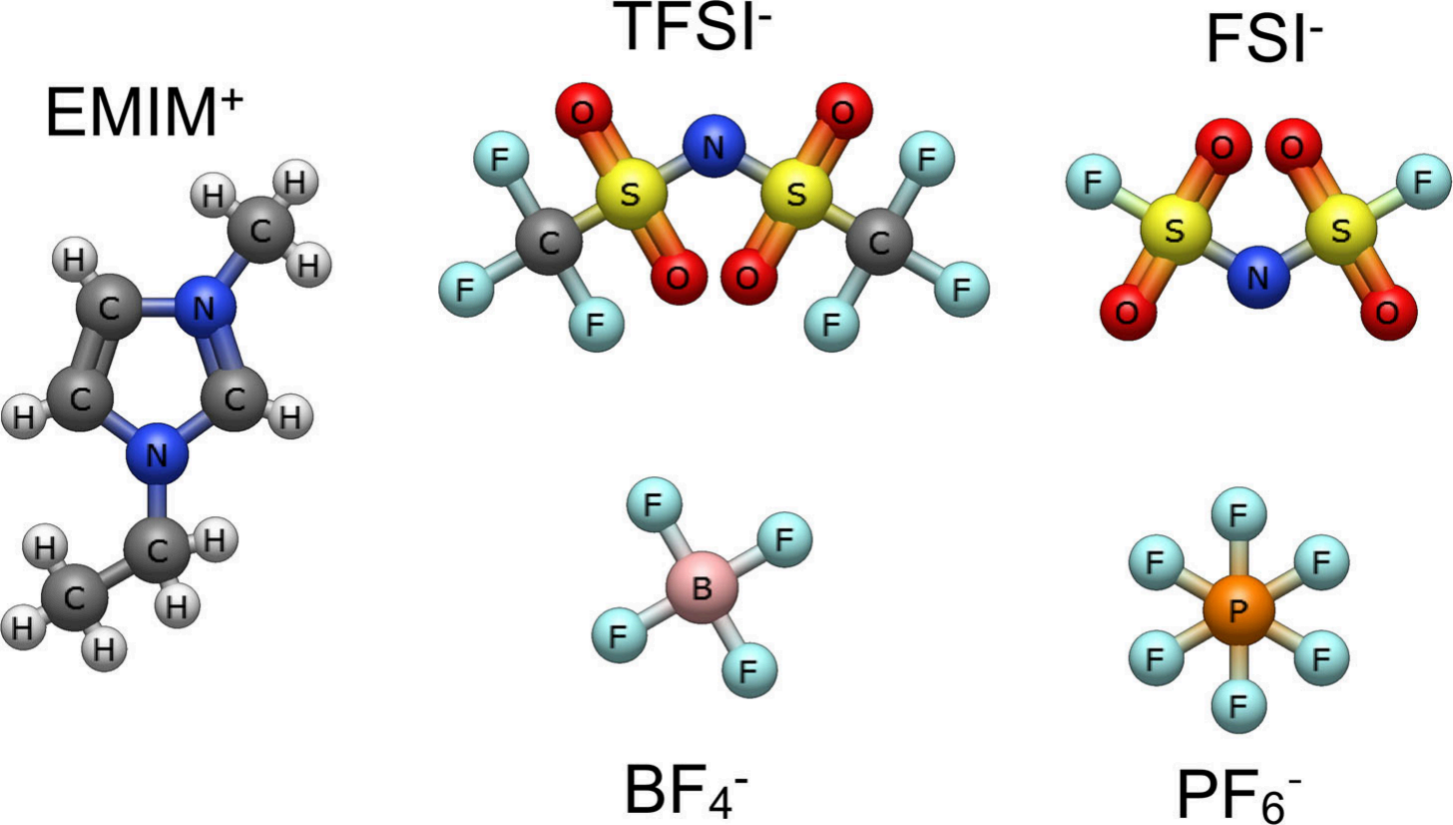
Structure of the 1-ethyl-3-methylimidazolium (EMIM^+^) cation and bis­(trifluoromethanesulfonyl)­imide (TFSI^–^), bis­(fluorosulfonyl)­imide (FSI^–^), hexafluorophosphate (PF_6_^–^), and tetrafluoroborate (BF_4_^–^) anions.

Figure 2 shows the NMRD profiles for ^1^H (a) and ^19^F (b) for all investigated ionic liquids. As observed in Figure 2a, the ^1^H relaxation rates decrease with increasing frequency. Among the samples, EMIM-BF_4_ (η = 38.208 mPa·s^36^) exhibits the highest relaxation rates across the entire frequency range, reflecting a slower molecular motion and higher viscosity. In contrast, EMIM-FSI (η = 22.28 mPa·s^37^) displays the lowest ^1^H relaxation rates, evidencing faster reorientational dynamics.

**Figure 2:**
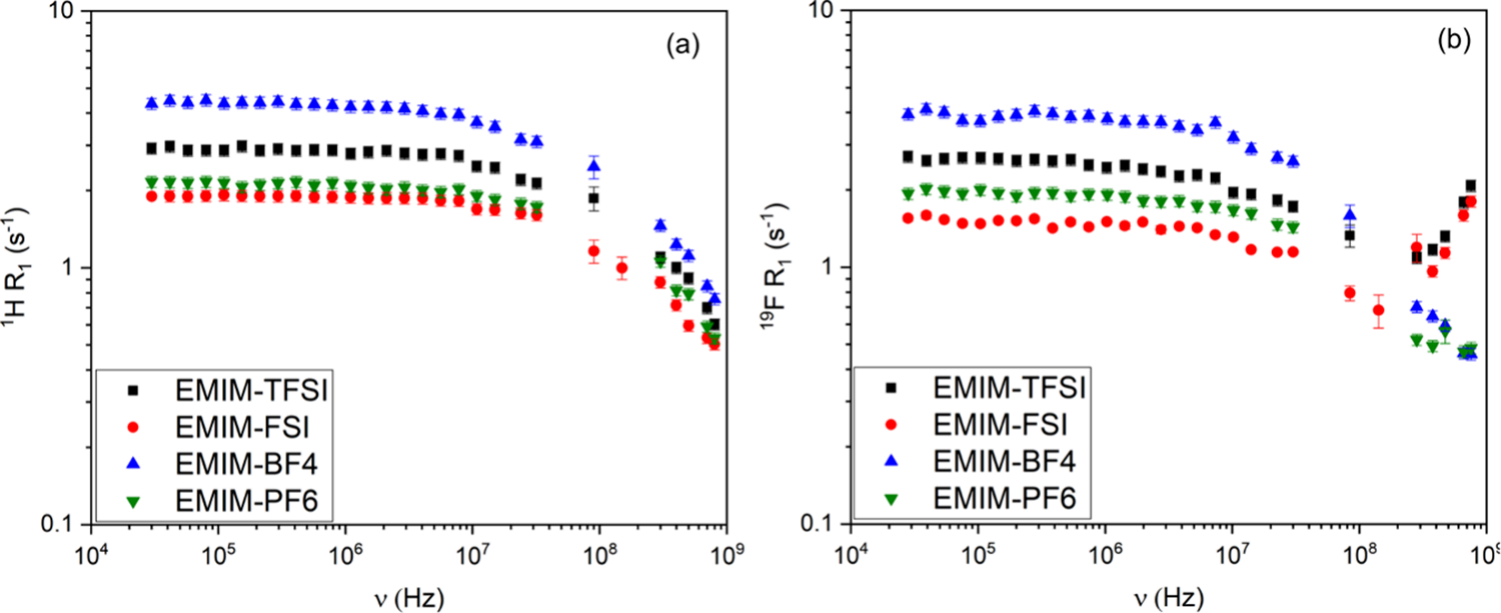
(a) ^1^H and (b) ^19^F relaxation rate (*R*_1_) dispersions for the ionic liquids studied.

For some of the systems, the ^19^F NMRD profiles in Figure 2b exhibit markedly different behaviors at higher frequencies. For BF_4_^–^, ^19^F *R*_1_ decreases with increasing frequency in a similar way as ^1^H *R*_1_. Contrarily, for the TFSI^–^ and FSI^–^ a remarkable increase of *R*_1_ is observed at high frequencies. Instead, PF_6_^–^ presents an intermediate behavior in comparison to the other ^19^F NMRD profiles of the other anions. This increase is not predicted by most relaxation models associated with the dipolar spin interaction, which typically predict a decrease in *R*_1_ with increasing frequency, as previously mentioned. One possibility could be that *R*_1_ increases at high magnetic fields due to paramagnetic enhancement relaxation effects.^38^ However, these enhancement effects typically manifest around tens of megahertz and certainly cannot be considered in the investigated systems that do not contain paramagnetic species. Indeed, this trend could instead arise from chemical shift anisotropy (CSA), which becomes increasingly relevant at high magnetic fields.^29^

To quantify the individual contributions to relaxation and provide a physically meaningful interpretation of the NMRD profiles, we made the following considerations. The rotational/reorientational motion of elongated and asymmetric ions, such as EMIM^+^ and TFSI^–^, was treated using Nordio’s model, which considers two distinct correlation times: one along the long molecular axis (τ_*z*_) and another along the short axis (τ_*x*_). This approach is particularly useful for describing the rotational reorientation of elongated and asymmetrical molecules, which lack well-defined intramolecular distances between specific spin pairs. Nordio’s rotational model (*R*_1_^Rot^) can be written as (eqs 1–4):^39–41^

$$R_{1}^{\mathrm{Rot}}\left(\omega_{i}\right)=\frac{3}{4}K_{\mathrm{dip}}^{ii}\left[J_{R}^{\left(1\right)}\left(\omega_{i}\right)+J_{R}^{\left(2\right)}\left(2\omega_{i}\right)\right]$$ 1

where ω_*i*_ = 2*πν*_*i*_ is the Larmor frequency of the spin *i* (in the present study ^1^H or ^19^F), *K*_dip_^*ii*^ is the dipolar coupling constant in the case of homonuclear interaction (eq 2). In general, *K*_dip_^*ij*^ is expressed as

$$K_{\mathrm{dip}}^{ij}=\frac{3}{2}{\left(\frac{\mu_{0}\gamma_{i}\gamma_{j}\hbar }{4\pi }\right)}^{2}$$ 2

Here, *ℏ* is Plank’s constant divided by 2π, μ_0_ is the vacuum magnetic permeability, and γ_*i*_ is the gyromagnetic ratio of the nuclei *i*

In the case of isotropic liquids, and absence of orientational order (*S* = 0), the spectral density function, *J*_*R*_^(*k*)^ (for *k* = 0,1,2) can be written as (eq 3)^42^

$$J_{R}^{\left(k\right)}\left(\omega \right)=\frac{4}{15}c_{k}\overset{2}{\underset{m=0}{\sum }}A^{\left(m\right)}\frac{\tau_{m}}{1+\tau_{m}^{2}\omega^{2}}$$ 3

where *c*_*k*_ = 6, 1, 4 for *k* = 0, 1, 2, respectively, and

$$A^{\left(m\right)}=\{\begin{array}{c} \overset{®}{{\left(3\cos^{2}\alpha_{\mathrm{ij}}-1\right)}^{2}/\left(4r_{\mathrm{ij}}^{6}\right)\;},m=0\\ \overset{®}{3\sin^{2}2\alpha_{\mathrm{ij}}/\left(4r_{\mathrm{ij}}^{6}\right)},m=1\\ \overset{®}{3\sin^{4}\alpha_{\mathrm{ij}}/\left(4r_{\mathrm{ij}}^{6}\right)},m=2 \end{array}$$ 4

Here, α_*ij*_ is the angle that each dipole spin vector makes with the main axis; and *r*_*ij*_ is the inter spin distance. τ_*m*_ depends on correlation times for rotation around the main (τ_*z*_) and short axis (τ_*x*_), and for *S* = 0 (absence of any orientational order)

$${\left(\tau_{m}\right)}^{-1}={\left(6\tau_{z}\right)}^{-1}\left[6+\left(\frac{\tau_{x}}{\tau_{z}}-1\right)m^{2}\right]$$ 5

In the case of spherical molecules, τ_*x*_ = τ_*z*_ = τ, and eq 3 with the use of eqs 4 and 5 can be simplified as eq 6:

$$J_{R}^{\left(k\right)}\left(\omega \right)=\frac{4}{15}c_{k}\frac{\tau }{1+\tau^{2}\omega^{2}}\left\langle \frac{1}{r_{ij}^{6}}\right\rangle$$ 6

which is equivalent to the classical and well-known Bloembergen–Purcell–Pound (BPP)^43^ spectral density function.

For the relaxation phenomenon associated with the translational self-diffusion of molecules, Torrey’s model describes the motion as random jumps in all directions. The homonuclear relaxation rate due to translational diffusion is given by^24^

$$R_{1_{ii}}^{\mathrm{SD}}\left(\omega_{i}\right)=\frac{3}{4}K_{\mathrm{dip}}^{ii}\left[J_{\mathrm{SD}}^{\left(1\right)}\left(\omega_{i}\right)+J_{\mathrm{SD}}^{\left(2\right)}\left(2\omega_{i}\right)\right]$$ 7

Here the spectral density *J*_SD_^(*k*)^(ω_*i*_) is expressed by eq 8:

$$J_{\mathrm{SD}}^{\left(k\right)}\left(\omega_{i}\right)=c_{k}\frac{n_{i}\tau_{D}}{d_{ii}^{3}}J\left(\omega_{i}\right)$$ 8

In eq 8, τ_*D*_ is the translational correlation time, *d*_*ii*_ is the average intermolecular spin distance, *D* is the self-diffusion coefficient, *r* is the average jump distance, and *n*_*i*_ is the density of spins of the nucleus of spin *i*. According to Torrey, the mean square displacement is given by eq 9:^22,44^

$$\left\langle r^{2}\right\rangle =6\tau_{D}D$$ 9

For the heteronuclear contribution, (*R*_1_*ij*__^*SD*^) is expressed as eq 10 ^9,29^

$$R_{1_{ij}}^{\mathrm{SD}}\left(\omega_{i}\right)=\frac{3}{4}K_{\mathrm{dip}}^{ij}\frac{n_{j}\tau_{D}}{d_{ij}^{3}}\left[\frac{1}{3}J\left(|\omega_{i}-\omega_{j}|\right)+J\left(\omega_{i}\right)+2J\left(\omega_{i}+\omega_{j}\right)\right],i\neq j$$ 10

When calculating relaxation contributions associated with translational diffusion, only intermolecular interactions are relevant. For intermolecular homonuclear contributions, cation–cation and anion–anion distances were considered to determine the average intermolecular spin distance *d*_*ii*_. For heteronuclear contributions, only cation–anion (counterion) distances (*d*_*ij*_) were used, as ^19^F nuclei are located exclusively in the anion and ^1^H nuclei in the cation. All distance values were obtained from molecular dynamics simulations, as described in the Supporting Information.

In addition to the dipolar relaxation mechanisms described above, fluctuations in the local magnetic field due to chemical shift anisotropy (CSA) can also contribute to spin relaxation, particularly for ^19^F nuclei. The contribution of chemical shift anisotropy (CSA) to the spin–lattice relaxation rate (*R*_1_^CSA^) for a ^19^F nucleus is given by eq 11:^28,35^

$$R_{1}^{\mathrm{CSA}}\left(\omega_{F}\right)=\frac{6}{40}\omega_{F}^{2}{\left(C_{\mathrm{SA}}\times 10^{-6}\right)}^{2}\frac{\tau_{F}}{1+{\left(\omega_{F}\tau_{F}\right)}^{2}}$$ 11

Here, *C*_SA_ represents the ^19^F shielding anisotropy in ppm, and τ_F_ is the correlation time associated with the fluctuation of the CSA tensor.

The fitting of the relaxation profiles was performed with the help of the online platform *fitteia*, which uses a nonlinear least-squares method to fit the relaxation models to the relaxation data.^45,46^ The total relaxation rate fitted to the experimental results is given by eq 12:

$$R_{1}^{i}\left(\omega_{i}\right)=R_{1}^{\mathrm{Rot}}\left(\omega_{i}\right)+R_{1_{ii}}^{\mathrm{SD}}\left(\omega_{i}\right)+R_{1_{ij}}^{\mathrm{SD}}\left(\omega_{i}\right)+{\left[R_{1}^{\mathrm{CSA}}\left(\omega_{F}\right)\right]}_{i=F}$$ 12

This model depends on the following parameters: *A* ^(*m*)^, τ_*z*_, τ_*x*_, *D*, *n*_*i*_, ⟨*r* ^2^⟩, *d*_*ij*,_ *d*_*ii*_, *C*_SA_, and τ_F_. In order to decrease the number of free parameters to obtain a consistent and reliable model fit, *A* ^(*m*)^, *D*, *n*_*i*_, *d*_*ij*_, and *d*_*ii*_, (Table 1) were obtained experimentally or estimated from molecular dynamics simulation as described in the Supporting Information. Note that the use of the measured self-diffusion coefficients *D* for cations (^1^H domain) and anions (^19^F domain) in the relaxation model automatically includes eventual ion association and correlated motions. Thus, the only unknown fitting parameters remaining were τ_*z*_, τ_*x*_, ⟨*r* ^2^⟩, *C*_SA_, and τ_*F*_. The fitting was performed within predefined, physically meaningful ranges derived from prior studies and theoretical considerations. The resulting best-fit parameters and their respective uncertainties are summarized in Table 2. These uncertainties were obtained considering the uncertainty of the experimental relaxation rates and fitting residues for χ^2^ normalized to 1.

**Table 1:** ^1^H and ^19^F Model Parameters Used as Fixed Values in the Model Fitting Procedures

Fitting Parameters	EMIM-TFSI	EMIM-FSI	EMIM-BF_4_	EMIM-PF_6_
*d*_HH_ (Å)^b^	8.9	8.7	7.3	8.4
*d*_FF_ (Å)^b^	9.5	8.5	7.0	7.5
*d*_HF_ (Å)^b^	5.0	4.7	4.3	4.6
*n*_H_ (m^–3^)^c^	2.58 × 10^28^	3.29 × 10^28^	4.30 × 10^28^	3.98 × 10^28^
*n*_F_ (m^–3^)^c^	1.41 × 10^28^	0.60 × 10^28^	1.56 × 10^28^	2.17 × 10^28^
*r*_FF_ (Å)^b^	–	2.7	2.3	2.3
*D*_H_ (m^2^/s)^a^	4.1 × 10^–11^	6.2 × 10^–11^	3.9 × 10^–11^	7.6 × 10^–11^
*D*_F_ (m^2^/s)^a^	2.4 × 10^–11^	4.6 × 10^–11^	3.0 × 10^–11^	5.4 × 10^–11^

a Obtained experimentally via PFG-NMR as described in the Supporting Information.

b Obtained by MD simulations, with representative values corresponding to the distance from the most intense peak in the RDF distribution, as described in the SI (Figures S2 and S3).

c Refers to the density of spins calculated from the molecular composition and density in Table S1 in the Supporting Information.

**Table 2:** Model Parameters Obtained for the Best Fit of Eq 12 to the ^1^H and ^19^F Experimental NMRD Data for the Studied Ionic Liquids

	τ_***z***_ (×10^–10^) s		τ_***x***_ (×10^–8^) s		*r* (×10^–10^) m		τ_F_ (×10^–10^)	*C*_SA_ (ppm)
	^1^H	^19^F	^1^H	^19^F	^1^H	^19^F	^19^F	^19^F
EMIM-TFSI	3.5 ± 0.6	9.0 ± 1.4	1.0 ± 0.2	2.1 ± 0.4	6.9 ± 0.4	6.9 ± 0.1	1.5 ± 0.3	69.5 ± 4.4
EMIM-FSI	1.8 ± 0.5	18 ± 0.9	0.77 ± 0.09	2.8 ± 0.6	7.9 ± 0.7	7.0 ± 0.1	3.1 ± 0.6	69.9 ± 4.2
EMIM-BF_4_	5.7 ± 0.7	1.0 ± 0.3^a^	1.16 ± 0.07	(0.010 ± 0.003)^a^	6.7 ± 0.1	7.6 ± 0.6	1.0 ± 0.3^a^	4.3 ± 0.4
EMIM-PF_6_	2.2 ± 0.3	0.7 ± 0.1^a^	0.7 ± 0.1	(0.007 ± 0.001)^a^	6.0 ± 1.5	7.8 ± 0.5	0.7 ± 0.1^a^	26.4 ± 2.6

a Values estimated for the condition τ_*z*_ = τ_*x*_ as explained in the main text.

Figure 3 shows the fitted frequency-dependent ^1^H relaxation rates for the studied ionic liquids. In all the frequency ranges, relaxation is dominated by molecular rotations, with correlation times along the main axis (τ_*z*_) reflecting the viscosity of the systems (c.a. EMIM-BF_4_ exhibited the slowest motion, in agreement with its higher viscosity). At lower frequencies (below 10 MHz), the translational relaxation mechanism becomes more relevant and the heteronuclear ^1^H–^19^F dipolar interaction serves as an efficient contributor to ^1^H relaxation, particularly in systems with higher ^19^F spin density such as EMIM-TFSI and EMIM-PF_6_. This is consistent with earlier reports showing that ^19^F can influence ^1^H relaxation.^21^

**Figure 3:**
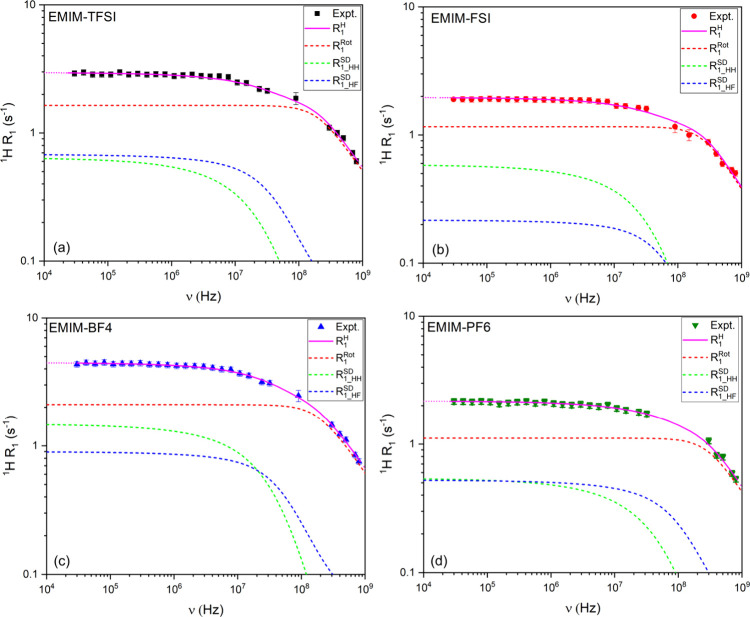
^1^H relaxation dispersion profiles of the studied ionic liquids: (a) EMIM-TFSI, (b) EMIM-FSI, (c) EMIM-BF_4_, and (d) EMIM-PF_6_.

Using Torrey’s stochastic approach to describe ^1^H spin relaxation by translational diffusion, we observed that the root mean-squared flight distance $\sqrt{\left\langle r^{2}\right\rangle }$ remains smaller than the average EMIM^+^–EMIM^+^ distance across all samples, as calculated by MD (Figure S2). This indicates that EMIM^+^ molecular motion is governed by random diffusive motion where the mean square jump distance is less than the average distance between molecular sites.^24^

Figure 4 displays the fitted ^19^F relaxation dispersion profiles. Unlike that of ^1^H, the high-field ^19^F relaxation behavior cannot be explained by dipolar interactions alone. For EMIM-TFSI and EMIM-FSI, the pronounced increase in *R*_1_ above 300 MHz is a clear signature of chemical shift anisotropy (CSA), with best-fit *C*_SA_ values of around 69 ppm. This CSA contribution dominates over dipolar relaxation and becomes the primary relaxation mechanism at high fields.

**Figure 4:**
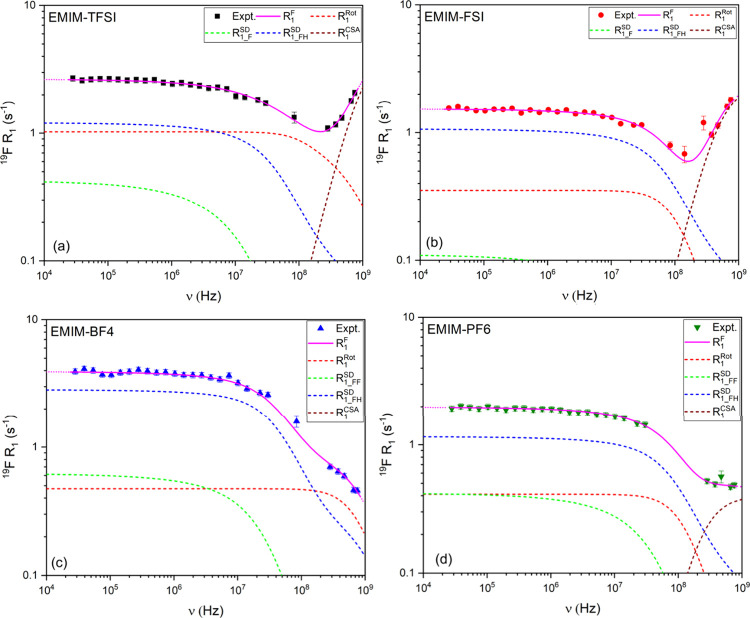
^19^F relaxation dispersion profiles of the studied ionic liquids: (a) EMIM-TFSI, (b) EMIM-FSI, (c) EMIM-BF_4_, and (d) EMIM-PF_6_.

The anion correlation times (Table 1) provide further insight into the dynamics. For the elongated and asymmetric anions TFSI^–^ and FSI^–^, τ_*z*_ is shorter in TFSI^–^ (9 × 10^–10^ s) than in FSI^–^ (18 × 10^–10^ s), likely due to the presence of a dominant reorientation axis associated with the *C*_3*v*_ symmetry of TFSI^–^, which facilitates more efficient modulation of the CSA tensor through faster rotational motion.

In contrast, the smaller and more symmetric anions PF_6_^–^ and BF_4_^–^, exhibit lower *C*_SA_ amplitudes (26 and 4 ppm, respectively), and the extracted correlation times vary: τ is relatively shorter for PF_6_^–^ (7 × 10^–11^ s), while BF_4_^–^ shows a longer τ (1.0 × 10^–10^ s). These results reflect differences not only in symmetry but also in size and local environment, highlighting that both the amplitude of *C*_SA_ and the time scale of molecular motion jointly determine the relaxation behavior. Notably, even in EMIM-PF_6_, the dipolar-only model fails to fully reproduce the high-field data, confirming that CSA, though weaker than that in TFSI^–^ or FSI^–^, still plays a measurable role. The *C*_SA_ values reported here (4–69 ppm) are consistent with those found in the literature for polycrystalline amino acids (10–75 ppm),^47,48^ confirming that they are physically meaningful. Additionally, CSA patterns have been experimentally observed for the FSI^–^ anion in organic ionic plastic crystal materials by solid-state NMR,^49^ further supporting its role as an efficient ^19^F relaxation mechanism, as described here.

The parameter *r* in the Torrey model reflects the root-mean-squared flight distance associated with translational diffusion that modulates the ^19^F dipolar interaction.^24^ For EMIM-BF_4_ and EMIM-PF_6_, the values of *r* (∼7.6–7.8 Å) are close to the distance between neighboring anions obtained by MD simulations (available in the SI, Table S2). In contrast, TFSI^–^ and FSI^–^ show slightly smaller *r* values (∼6.9–7.0 Å) than the corresponding anion–anion distances. This deviation may be related to differences in the anion structure and dynamics that affect the effective displacement characteristic of the translational relaxation mechanism.

This work experimentally provides a comprehensive analysis of ^1^H and ^19^F relaxation mechanisms in EMIM-based ionic liquids, relaxation dispersion covering a broad range of magnetic fields with model-based fitting constrained by diffusion and structural data. For the first time, we demonstrate that chemical shift anisotropy (CSA) plays a dominant role in governing the ^19^F relaxation behavior of fluorinated anions in ionic liquids at high magnetic fields. This relaxation mechanism has often been overlooked. Although CSA effects are averaged out in the spectra of isotropic liquids due to fast molecular motion, the time-dependent fluctuations of the CSA tensor remain a significant and efficient relaxation pathway—particularly for anisotropic molecules at high magnetic fields. In particular, the significant increase in ^19^F *R*_1_ for TFSI^–^ and FSI^–^ is shown to arise from CSA contributions (∼69 ppm), which exceed the expected dipolar contributions and dominate the overall relaxation behavior at high magnetic fields. These findings complement the general interpretation of ^19^F relaxation by dipolar interactions and underscore the importance of explicitly accounting for the CSA when analyzing ^19^F dynamics in fluorinated systems. Our results also reinforce the relevance of intermolecular contributions to relaxation at low frequencies through translational diffusion and apply an interpretation that uses the mean-squared jump distance as the only adjustable parameter, while diffusivities and intermolecular distances are known from experiments or simulations. The good agreement between the experimental data and the fitting curves across the full frequency range serves as a self-consistent proof of the reliability of the relaxation model used to describe the observed *R*_1_ profiles. Consequently, any attempt to deconvolute model deviations due to the presence of ion pairs would remain inconclusive. Together, these insights provide new guidelines for interpreting ^19^F NMR data in fluorine-containing ions and highlight the potential of CSA-sensitive nuclei in the rational design of ionic liquid-based materials.

## Supplementary Material





## Notes

Additional experimental details (self-diffusion measurements, longitudinal relaxation), MD simulations, and frequency-dependent R1 relaxation data (PDF)
Transparent Peer Review report available (PDF)
