# Supplementary material for: Expanded View of NMR Spin–Lattice Relaxation in Fluorine-Containing Ionic Liquids
Source: J Phys Chem Lett. 2025 Jul 21;16(30):7622–9. doi: 10.1021/acs.jpclett.5c01665 (PMC12319907; doi:10.1021/acs.jpclett.5c01665)
Supplement: Supplementary file 1 [file jz5c01665_si_001.pdf]

Supplementary Material to:

An expanded view of NMR spin-lattice relaxation in fluorine-containing ionic liquids

Giselle de Araujo Lima e Souza[*†], Elizabeth Brandwein[§], Emilia Pelegano-Titmuss[†], Phillip Stallworth[†], Yong Zhang[‡], Pedro José de Oliveira Sebastião[¶], Steven Greenbaum[†]*

†Department of Physics, Hunter College, CUNY, New York - USA

*giselle.souza@hunter.cuny.edu

[§]Department of Chemistry, New York City College of Technology, New York - USA

[§]Department of Chemical and Biomolecular Engineering, University of Notre Dame, Indiana - USA

[¶]CeFEMA and Department of Physics, Instituto Superior Técnico, Universidade de Lisboa, Lisboa - Portugal

1. Self-diffusion measurements

Self-diffusion coefficients were measured using Pulsed Field Gradient Nuclear Magnetic Resonance (PFG NMR) at 293 K for EMIM-TFSI, EMIM-FSI, and EMIM-BF_4, and at 338 K for EMIM-PF_6. Diffusion of the $EMIM^+$ cation and each anion was independently measured in the ^1H and ^{19}F frequency domains using the bipolar pulsed gradient stimulated echo (BPP-LED) pulse sequence.

Each experiment consisted of 8 transients per gradient step. The spectral widths were set to 12 ppm for ^1H and between 10–40 ppm for ^{19}F, depending on the chemical shift range of the anion. A relaxation delay of at least five times the T_1 value was used, and 8 dummy scans were applied before acquisition to ensure steady-state conditions.

Gradient amplitudes were linearly incremented from 2% to 95% of the maximum gradient strength over 16 steps. The gradient pulse duration (δ) and diffusion time (Δ) were optimized for each system to achieve approximately 95% signal attenuation for the slowest-diffusing species at the highest gradient value. The measurements were carried out on a Bruker Avance 400 MHz spectrometer (9.4 T) equipped with a 5 mm z-gradient BBFO iProbe. Gradient durations (δ) ranged from 3 to 6 ms, and diffusion times (Δ) were between 0.1 and 0.2 s. The maximum gradient strength was 50 $G \cdot cm^{-1}$.

Spectral processing included manual phasing and automatic baseline correction. An exponential line broadening of 0.3 Hz was applied in the F2 dimension. Diffusion coefficients (D) were extracted by fitting the attenuation of signal intensity to the Stejskal–Tanner equation [1] (Eq. S1):

$$\frac{I}{I_0} = exp\left(-\gamma^2 g^2 \delta^2 D \left(\Delta - \frac{\delta}{3}\right)\right) \quad \text{(S1)}$$

where I and I_0 are the intensity with and without field gradient, γ is the gyromagnetic ratio, g and δ are the gradient strength and duration, and Δ is the diffusion time.

2. Longitudinal Relaxation NMR

For FFC NMR experiments, samples were transferred into 10 mm NMR tubes and flame-sealed to prevent contamination. Measurements were performed using a Spinmaster FFC2000 CDC Relaxometer (Stelar, Italy) with standard pre-polarized (PP) or non-polarized (NP) pulse sequences. Relaxation dispersion curves were recorded over a relaxation field (B_R) range of 30 kHz to 32 MHz for ^1H, 28 kHz to 30 MHz for ^{19}F. The polarization field (B_P) was set to 15 MHz for ^1H and ^{19}F, with a field slew rate of 13 $MHz \cdot ms^{-1}$ (all in terms of the ^1H Larmor frequency) and a switching time of 3 ms. Signal scans (4–16) were acquired for 8 delay (τ) values at each frequency. T_1 was calculated from the magnetization (M) recovery curves relative to τ by fitting a mono-exponential equation (Eq. S2).

$$M_z(\tau) = M_0(B_R) + [M_0(B_P) - M_0(B_R)] \exp(-\tau/T_1(B_R)) \quad \text{(S2)}$$

$R_1=1/T_1$ relaxation rates were also measured using conventional NMR techniques using various spectrometers: a 90 MHz permanent magnet (Anasazi Instruments), a 300 MHz Varian NMR spectrometer, and 400 MHz, 500 MHz, 700 MHz and 800 MHz Bruker spectrometers. The *inversion-recovery* pulse sequence was used, and data was acquired with a list of 16-32 delay times (τ). For EMIM-FSI, additional T_1 measurements were performed in the fringe (non-uniform) field of a 300 MHz magnet on a Varian spectrometer. The probe was physically lowered within the magnet bore to access lower magnetic fields, corresponding to 149.5 MHz for ^{1}H and 141.2 MHz for ^{19}F. High power rf pulses were used to ensure spectral coverage of the broad lines resulting from field inhomogeneity. A saturation recovery spin-echo sequence was employed (see Fig. S1), consisting of 50 π saturation pulses spaced by Δ = 300 ms (total saturation time = 15 s), followed by a variable recovery delay τ (0.01–20 s) and a spin-echo acquisition. The delay between the $\pi/2$ and π pulses was set to τ_{echo} = 75 µs, and signal acquisition occurred after the π pulse. T_1 values were extracted from the echo intensity as a function of the recovery delay τ. A recycle delay of D_1 = 500 ms was applied between scans.

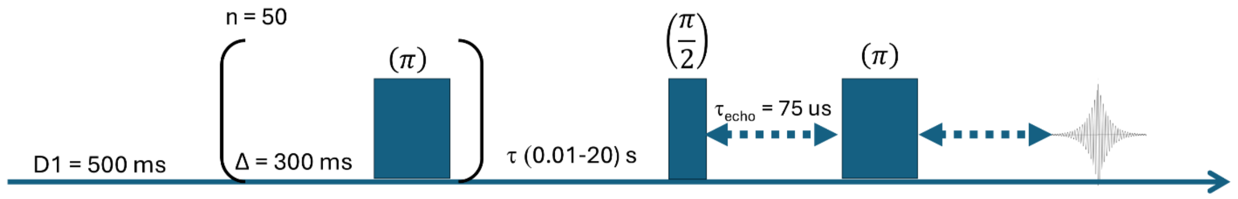

Figure S1: Pulse sequence used for T_1 measurements via saturation recovery with echo acquisition.

Relaxation times were calculated by integrating the raw data and fitting the signal intensity decay using *T1/T2* module from TopSpin for Bruker experiments and *Origin 2019b* for others.

3. Molecular Simulations

Molecular dynamics (MD) simulations were carried out using the package LAMMPS[2]. For each ionic liquid (IL), the simulation box was built by placing the appropriate number (see Table S1) of cation and anion ions randomly in a cubic box using the package Packmol [3,4]. Each system was then equilibrated for 5 ns in the isothermal-isobaric (NPT) ensemble to determine the density, followed by a 20 ns production simulation in the canonical ensemble (NVT) with the atomic coordinates saved every 500 ps. The Nosé-Hoover thermostat[5] and the extended Lagrangian approach [6] were applied to control the temperature and pressure, respectively. A time constant of 100 fs was used in both the thermostat and barostat. The pressure was fixed at one atmosphere in all constant pressure simulations with isotropic volume fluctuations and the temperature was set to be 338 K for [EMIM][PF_6] and 293 K for the other ILs (see Table S1), consistent with experiments. A timestep of 1 fs was used in all simulations.

The general Amber force field (GAFF)[7] was used to describe the interactions. To derive partial atomic charges used with GAFF, electronic structure calculation was carried out on each isolated ion at the B3LYP/6-311++g(d,p) level using the package Gaussian.[8] The atomic charges were then derived based on the optimized structure by fitting the electrostatic potential surface obtained from this calculation using the restrained electrostatic potential (RESP) method[9]. To match the experimental density (see Table S1), the partial charges of [EMIM][TFSI] and [EMIM][FSI] were scaled uniformly by 0.8 whereas the full charges (±1 e) were used for [EMIM][PF_6] and [EMIM][BF_4]. The long-range electrostatic interactions were calculated using the particle-particle particle-mesh (PPPM) method[10] with a real space cutoff of 12 Å. The same cutoff was used for van der Waals interactions and a tail correction[10] was applied.

Table S1. The number of ion pairs in the simulation box, absolute value of the total charge on each ion, simulation temperature (T), calculated density, and the experimental density of each ionic liquid studied in the current work.

IL	# of ion pairs	Total charge (e)	T (K)	Density (Calc.)	Density (Exp.)
[EMIM][TFSI]	350	0.8	293	1.526	1.522 [11]
[EMIM][FSI]	400	0.8	293	1.458	1.446 [12]
[EMIM][PF_6]	500	1.0	338	1.431	1.435 [13]
[EMIM][BF_4]	500	1.0	293	1.277	1.287 [14]

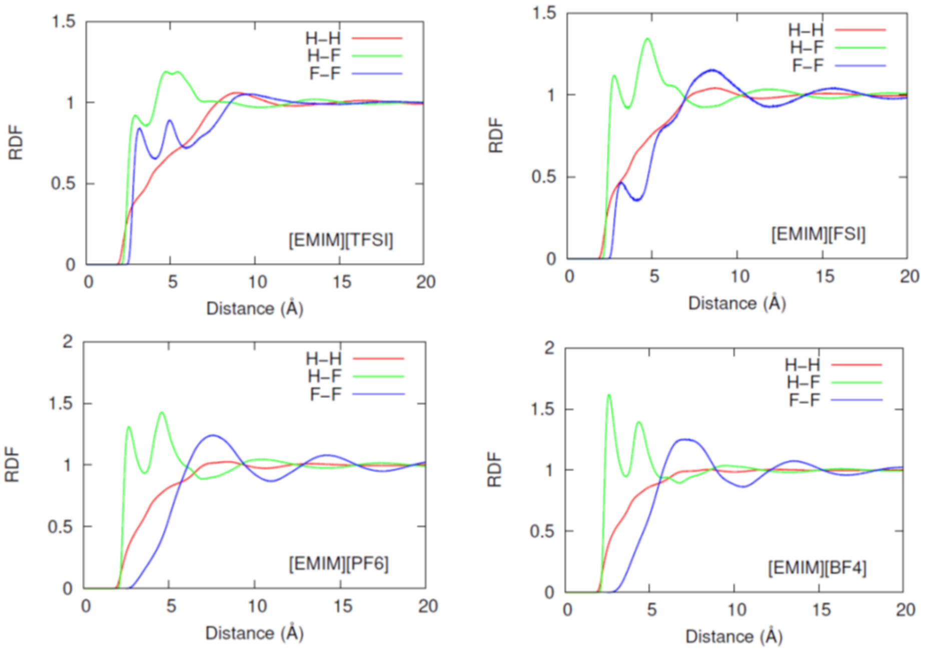

Figure S2: Calculated intermolecular radial distribution functions (RDFs) for each ionic liquid based on MD simulations.

Table S2: Intervals of intermolecular distances (in Å) based on MD simulations.

	EMIM-BF_4			EMIM-PF_6			EMIM-TFSI			EMIM-FSI		
	min	peak	max	min	peak	max	min	peak	max	min	peak	max
H-H	1.7	7.3	10.0	1.8	8.4	10.6	1.7	8.9	12.2	1.7	8.7	11.4
H-F	2.0	2.6	3.6	1.9	2.7	3.5	2.0	2.9	3.5	2.1	2.8	3.6
	3.6	4.3	6.7	3.5	4.6	7.0	3.5	5.0	7.0	3.6	4.7	8.1
F-F	2.6	7.0	10.5	2.5	7.5	11.0	2.4	3.2	4.1	2.4	3.3	4.1
							4.1	5.0	6.0	4.1	8.5	12.0
							6.0	9.5	14.8			

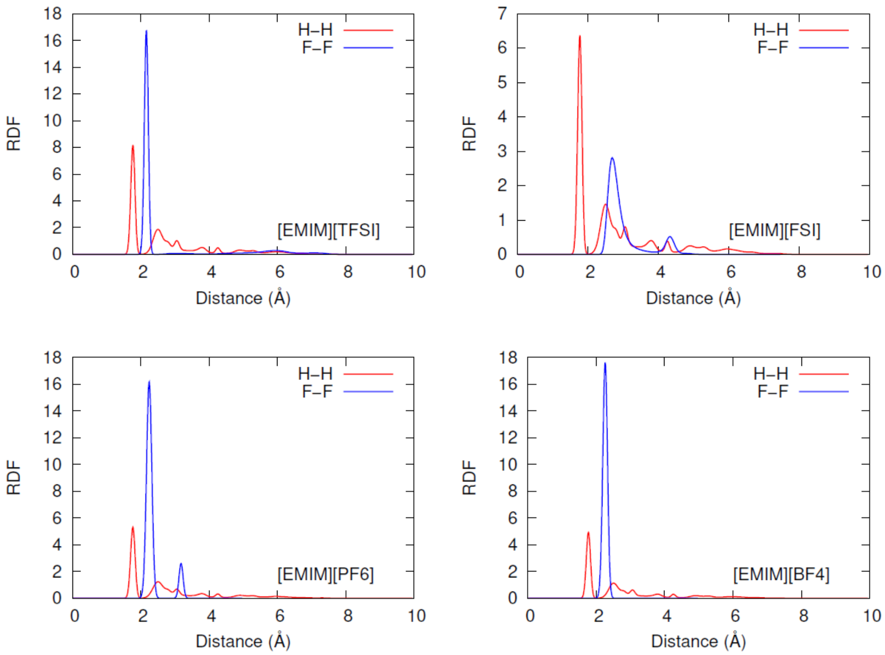

Figure S3: Calculated intramolecular radial distribution functions (RDFs) for each ionic liquid based on MD simulations.

Table S3: Intervals of intramolecular distances (in Å) based on MD simulations.

	EMIM-BF_4			EMIM-PF_6			EMIM-TFSI			EMIM-FSI		
	min	peak	max	min	peak	max	min	peak	max	min	peak	max
H-H	1.4	1.8	2.0	1.4	1.8	2.0	1.4	1.8	2.0	1.4	1.8	2.0
	2.0	2.5	2.9	2.0	2.5	2.9	2.0	2.5	2.9	2.0	2.5	2.9
	2.9	3.1	3.5	2.9	3.1	3.4	2.9	3.1	3.5	2.9	3.1	3.4
F-F	1.9	2.3	2.5	1.9	2.3	2.6	1.9	2.2	2.4	2.3	2.7	3.9
				2.6	3.2	3.4				3.9	4.3	4.7

Table S4: Frequency-dependent R_1 relaxation data for ^1H nucleus in EMIM-based ionic liquids measured at 20 °C.

	EMIM-FSI		EMIM-TFSI		EMIM-BF_4		EMIM-PF_6	
ν (Hz)	R_1 (s^{-1})	± error (s^{-1})	R_1 (s^{-1})	± error (s^{-1})	R_1 (s^{-1})	± error (s^{-1})	R_1 (s^{-1})	± error (s^{-1})
8.00E+08	0.504	0.025	0.602	0.030	0.755	0.038	0.533	0.027
7.00E+08	0.534	0.027	0.698	0.035	0.848	0.042	0.591	0.030
5.00E+08	0.596	0.030	0.910	0.046	1.113	0.056	0.791	0.040
4.00E+08	0.715	0.036	1.000	0.050	1.233	0.062	0.817	0.041
3.00E+08	0.880	0.044	1.100	0.055	1.458	0.073	1.059	0.053
1.50E+08	1.000	0.100	-	-	-	-	-	--
9.00E+07	1.162	0.120	1.866	0.200	2.472	0.250	-	-
3.20E+07	1.604	0.080	2.133	0.107	3.092	0.155	1.726	0.086
2.40E+07	1.630	0.082	2.204	0.110	3.160	0.158	1.779	0.089
1.50E+07	1.682	0.084	2.456	0.123	3.538	0.177	1.843	0.092
1.08E+07	1.692	0.085	2.482	0.124	3.684	0.184	1.905	0.095
7800000	1.824	0.091	2.727	0.136	3.947	0.197	2.025	0.101
5620000	1.829	0.091	2.776	0.139	3.974	0.199	1.974	0.099
4050000	1.865	0.093	2.757	0.138	4.078	0.204	2.009	0.100
2920000	1.869	0.093	2.786	0.139	4.169	0.208	2.058	0.103
2110000	1.869	0.093	2.856	0.143	4.209	0.210	2.024	0.101
1520000	1.868	0.093	2.821	0.141	4.228	0.211	2.049	0.102
1100000	1.880	0.094	2.781	0.139	4.229	0.211	2.083	0.104
790000	1.889	0.094	2.869	0.143	4.291	0.215	2.142	0.107
570000	1.889	0.094	2.878	0.144	4.326	0.216	2.093	0.105
410000	1.907	0.095	2.856	0.143	4.336	0.217	2.159	0.108
296000	1.899	0.095	2.901	0.145	4.433	0.222	2.137	0.107
213000	1.901	0.095	2.861	0.143	4.394	0.220	2.105	0.105
154000	1.907	0.095	2.974	0.149	4.402	0.220	2.067	0.103
111000	1.919	0.096	2.858	0.143	4.358	0.218	2.146	0.107
80200	1.907	0.095	2.865	0.143	4.488	0.224	2.164	0.108
57700	1.896	0.095	2.859	0.143	4.395	0.220	2.143	0.107
41700	1.902	0.095	2.964	0.148	4.468	0.223	2.158	0.108
30000	1.896	0.012	2.914	0.146	4.345	0.217	2.160	0.108

Table S5: Frequency-dependent R_1 relaxation data for ^{19}F nucleus in EMIM-based ionic liquids measured at 20 °C.

	EMIM-FSI		EMIM-TFSI		EMIM-BF_4		EMIM-PF_6	
ν (Hz)	R_1 (s^{-1})	± error (s^{-1})	R_1 (s^{-1})	± error (s^{-1})	R_1 (s^{-1})	± error (s^{-1})	R_1 (s^{-1})	± error (s^{-1})
7.53E+08	1.804	0.090	2.075	0.104	0.458	0.023	0.485	0.024
6.59E+08	1.591	0.080	1.789	0.089	0.462	0.023	0.470	0.024
4.71E+08	1.137	0.057	1.320	0.066	0.593	0.030	0.564	0.060
3.76E+08	0.964	0.048	1.170	0.059	0.644	0.032	0.493	0.025
2.82E+08	1.194	0.150	1.090	0.055	0.700	0.035	0.522	0.026
1.41E+08	0.680	0.100	-	-	-	-	-	-
8.40E+07	0.794	0.053	1.328	0.130	1.587	0.160	-	-
3.01E+07	1.148	0.022	1.722	0.086	2.580	0.129	1.435	0.072
2.26E+07	1.144	0.009	1.822	0.091	2.674	0.134	1.467	0.073
1.41E+07	1.171	0.016	1.922	0.096	2.889	0.144	1.620	0.081
1.02E+07	1.310	0.031	1.953	0.098	3.205	0.160	1.669	0.083
7340000	1.337	0.025	2.225	0.111	3.654	0.183	1.719	0.086
5290000	1.424	0.034	2.284	0.114	3.418	0.171	1.730	0.087
3820000	1.444	0.020	2.262	0.113	3.536	0.177	1.811	0.091
2750000	1.402	0.051	2.359	0.118	3.680	0.184	1.803	0.090
1980000	1.500	0.011	2.409	0.120	3.692	0.185	1.814	0.091
1430000	1.449	0.041	2.483	0.124	3.694	0.185	1.885	0.094
1030000	1.503	0.022	2.443	0.122	3.788	0.189	1.916	0.096
743296	1.435	0.035	2.500	0.125	3.887	0.194	1.922	0.096
536003	1.500	0.033	2.616	0.131	3.851	0.193	1.895	0.095
386205	1.419	0.030	2.590	0.130	3.962	0.198	1.949	0.097
278583	1.545	0.031	2.623	0.131	4.056	0.203	1.950	0.098
200791	1.517	0.025	2.595	0.130	3.918	0.196	1.893	0.095
144980	1.521	0.027	2.643	0.132	3.855	0.193	1.942	0.097
104649	1.477	0.011	2.666	0.133	3.709	0.185	2.002	0.100
75481.4	1.480	0.026	2.669	0.133	3.727	0.186	1.932	0.097
54264.6	1.530	0.024	2.646	0.132	4.008	0.200	1.975	0.099
39250.1	1.591	0.050	2.598	0.130	4.121	0.206	2.018	0.101
28203.7	1.551	0.036	2.694	0.135	3.932	0.197	1.934	0.097

References

(1) Stejskal, E. O.; Tanner, J. E. Spin Diffusion Measurements: Spin Echoes in the Presence of a Time-Dependent Field Gradient. *The Journal of Chemical Physics* **1965**, *42* (1), 288–292. https://doi.org/10.1063/1.1695690.

(2) Plimpton, S. Fast Parallel Algorithms for Short-Range Molecular Dynamics. *J Comput Phys* **1995**, *117* (1), 1–19. https://doi.org/10.1006/jcph.1995.1039.

(3) Martínez, L.; Andrade, R.; Birgin, E. G.; Martínez, J. M. P <scp>ACKMOL</Scp> : A Package for Building Initial Configurations for Molecular Dynamics Simulations. *J Comput Chem* **2009**, *30* (13), 2157–2164. https://doi.org/10.1002/jcc.21224.

(4) Martínez, J. M.; Martínez, L. Packing Optimization for Automated Generation of Complex System's Initial Configurations for Molecular Dynamics and Docking. *J Comput Chem* **2003**, *24* (7), 819–825. https://doi.org/10.1002/jcc.10216.

(5) Hoover, W. G. Canonical Dynamics: Equilibrium Phase-Space Distributions. *Phys Rev A (Coll Park)* **1985**, *31* (3), 1695–1697. https://doi.org/10.1103/PhysRevA.31.1695.

(6) Shinoda, W.; Shiga, M.; Mikami, M. Rapid Estimation of Elastic Constants by Molecular Dynamics Simulation under Constant Stress. *Phys Rev B* **2004**, *69* (13), 134103. https://doi.org/10.1103/PhysRevB.69.134103.

(7) Wang, J.; Wolf, R. M.; Caldwell, J. W.; Kollman, P. A.; Case, D. A. Development and Testing of a General Amber Force Field. *J Comput Chem* **2004**, *25* (9), 1157–1174. https://doi.org/10.1002/jcc.20035.

(8) Frisch, M. J.; Trucks, G.; Schlegel, H. B.; Scuseria, G. E.; Robb, M. A.; Cheeseman, J.; Scalmani, G.; Barone, V.; Mennucci, B.; Petersson, G. A.; Nakatsuji, H.; Caricato, M.; Li, X.; Hratchian, H. P.; Izmaylov, A. F.; Bloino, J.; Zheng, G.; Sonnenberg, J.; Hada, M.; Fox, D. Gaussian 09 Revision A.1. Gaussian Inc. **2009**.

(9) Bayly, C. I.; Cieplak, P.; Cornell, W.; Kollman, P. A. A Well-Behaved Electrostatic Potential Based Method Using Charge Restraints for Deriving Atomic Charges: The RESP Model. *J Phys Chem* **1993**, *97* (40), 10269–10280. https://doi.org/10.1021/j100142a004.

(10) Hockney, R.; Eastwood, J. *Computer Simulation Using Particles*; Adam Hilger, New York, 1989.

(11) Jacquemin, J.; Husson, P.; Majer, V.; Costa Gomes, M. F. Influence of the Cation on the Solubility of CO2 and H2 in Ionic Liquids Based on the

Bis(Trifluoromethylsulfonyl)Imide Anion. *J Solution Chem* **2007**, *36* (8), 967–979. https://doi.org/10.1007/s10953-007-9159-9.

(12) Yamamoto, T.; Matsubara, R.; Nohira, T. Highly Conductive Ionic Liquid Electrolytes for Potassium-Ion Batteries. *J Chem Eng Data* **2021**, *66* (2), 1081–1088. https://doi.org/10.1021/acs.jced.0c00879.

(13) Taguchi, R.; Machida, H.; Sato, Y.; Smith, R. L. High-Pressure Densities of 1-Alkyl-3-Methylimidazolium Hexafluorophosphates and 1-Alkyl-3-Methylimidazolium Tetrafluoroborates at Temperatures from (313 to 473) K and at Pressures up to 200 MPa. *J Chem Eng Data* **2009**, *54* (1), 22–27. https://doi.org/10.1021/je800224k.

(14) Neves, C. M. S. S.; Kurnia, K. A.; Coutinho, J. A. P.; Marrucho, I. M.; Lopes, J. N. C.; Freire, M. G.; Rebelo, L. P. N. Systematic Study of the Thermophysical Properties of Imidazolium-Based Ionic Liquids with Cyano-Functionalized Anions. *J Phys Chem B* **2013**, *117* (35), 10271–10283. https://doi.org/10.1021/jp405913b.
