# Supplementary material for: Expanded View of NMR Spin–Lattice Relaxation in Fluorine-Containing Ionic Liquids
Source: J Phys Chem Lett. 2025 Jul 21;16(30):7622–9. doi: 10.1021/acs.jpclett.5c01665 (PMC12319907; doi:10.1021/acs.jpclett.5c01665)
Supplement: Supplementary file 2 [file jz5c01665_si_002.pdf]

jz-2025-01665r.R1

Name: Peer Review Information for "An expanded view of NMR spin-lattice relaxation in fluorine-containing ionic liquids"

First Round of Reviewer Comments

Reviewer: 1

Comments to the Author

This manuscript presents a rigorous and timely study on NMR spin-lattice relaxation mechanisms in fluorine-containing ionic liquids (ILs), with particular focus on the underappreciated role of chemical shift anisotropy (CSA) in ^{19}F relaxation at high magnetic fields (>300 MHz). The work's major strengths include: (1) compelling experimental evidence challenging the traditional dipolar-dominance paradigm, especially for $TFSI^-$ and FSI^- anions showing significant CSA contributions (~69 ppm); (2) sophisticated methodology combining FFC-NMR (30 kHz-800 MHz), PFG-NMR, and MD simulations to constrain models; and (3) thoughtful application of Nordio's (anisotropic rotation) and Torrey's (diffusion) theories.

While the study is fundamentally sound and novel, minor revisions would strengthen it further: (a) explicit discussion of error propagation in CSA fitting and model limitations (e.g., transient ion pairing effects on diffusion); (b) improved data presentation through annotated figures (e.g., labeled molecular structures) and inclusion of raw NMRD data in SI; (c) deeper contextualization of CSA values versus literature and broader implications for IL design.

The work represents a significant advance in understanding IL dynamics and will be valuable for researchers using high-field NMR. With these revisions - primarily focused on clarifying uncertainties, enhancing readability, and expanding discussion - the manuscript will be ready for publication. I recommend minor revisions with high confidence in the study's importance to the field.

Reviewer: 2

Comments to the Author

This article presents careful measurements and a theoretically driven fitting approach to assessing the different spin magnetization/motional effects that influence T1 relaxation in some key representative ionic liquids (ILs). Using NMR T1 relaxation dispersion is a key experimental method for understanding how ILs translate and rotate. This dynamical information helps drive the uses of ILs in many applications including battery electrolytes and carbon capture. The key insight here is that the chemical shift anisotropy (CSA) for 19F has a strong effect on the 19F relaxation rates, and that this CSA effect provides new and systematic information about the rotations and translations of fluorinated anions. The authors wisely study the common series BF4-, PF6-, TFSI-, and FSI- and find very useful motional insights. This article is written clearly and should be published in JPC Letters. Below are a number of points that the authors should address before publication.

1) On the bottom of p. 2 in the sentence that ends with ref 6, I suggest using “T1” in the definition of NMRD and R1. For uninitiated readers, this might make them more enticed to read as many more physical chemists have heard of T1 than R1.

2) In the middle of p. 3, you state that the BPP and Cole Davidson models “…considered in the context of small and symmetric molecules, it relies on the assumption of an isotropic reorientation and a single averaged correlation time.” At least for BPP, this also relies an another huge assumption - that the coupled spins are covalently bonded to the same molecule, and/or have a well-defined distance between specific pairs of nuclei during the timescale of the experiment. This distance is definitely never well defined for ILs, and indeed the idea that NMR T1 or NOE can report on distances has been challenged based on distant dipolar field effects. There is a paper by H. Weingartner on this around 2010-2014, but I cannot find it at the moment. It is also well known, although rarely spoken, that distances obtained from T1 relaxivities on MRI contrast agents are only for relative comparison purposes since the BPP theory is violated (no proximity of a given water to a given agent molecule during the T1 measurement) for every case of water coordinating with a contrast agent. I encourage you to rewrite some of your statements about distances in the paper according to these concepts. Heteronuclear T1 effects necessarily involve only intermolecular terms in the case of these ILs, so the information

from these T1 terms is particularly suspect. These ideas relate to your values in Tables 1 and 2.

3) I applaud your use of the anisotropic rotation theory (originally from liquid crystal NMR) to assess relaxation contributions for FSI and TFSI. These are anisotropic molecules, even though their D and CSA anisotropy terms are averaged out in the spectra (well stated!). This aspect seems to be novel and exciting, and could perhaps be emphasized more in the abstract or intro? Also, you may be interested to look at a ref involving anisotropic spin interactions in FSI- in an OIPC (https://doi.org/10.1021/acs.jpcb.7b02780, authors L. Jin… J. Pringle, JPCB, 2017). In the OIPC, the CSA terms/patterns do not average out and do appear in the spectra!

4) In a few places where named models are mentioned (BPP, Cole Davidson, Nordio), key original references are left out, or only included much later in the paper. Please include the basic/original refs when the models/names are first mentioned.

5) I have a personal problem with the idea of a jump distance in liquid systems (with usually continuously varying jump distances), but you have written/applied the relevant model in as appropriate form as possible. I find it encouraging that the jump distances you extract from the R1 fits are pretty close to the anion-anion distances and other relevant average lengths in these systems. This relatively close matching usually does not come out of similar model fits when finding tau_D using dielectric spectroscopy or dielectric combined with NMR diffusion coeffs.

6) The derivations on p. 9 (eqs. 7-11) include some text that does not transition well to the next equation. Please consider rewriting this section to add more steps or more clearly delineate which equations go with which others. For example, when going from eq. 10 to 11, I originally thought you were determining 11 from 10, but it looks like you are simply introducing 11 from a reference and it is independent of 10.

7) Reference 16 appears to be incomplete.

8) Suggested rewriting of last sentence of main paper: "…and highlight the potential to use information from CSA-sensitive nuclei to bolster the rational design of ionic liquid-based materials."

Author's Response to Peer Review Comments:

Responses to Reviewers' Comments:

We thank the Reviewers for their careful evaluation and constructive suggestions. Below we provide a detailed point-by-point response to each comment. The authors' replies are shown in blue, while the original comments in black regular font. An annotated version of the manuscript with changes highlighted in yellow is also attached.

Reviewer: 1

Comments:
This manuscript presents a rigorous and timely study on NMR spin-lattice relaxation mechanisms in fluorine-containing ionic liquids (ILs), with particular focus on the underappreciated role of chemical shift anisotropy (CSA) in ^{19}F relaxation at high magnetic fields (>300 MHz). The work's major strengths include: (1) compelling experimental evidence challenging the traditional dipolar-dominance paradigm, especially for TFSI$^-$ and FSI$^-$ anions showing significant CSA contributions (~69 ppm); (2) sophisticated methodology combining FFC-NMR (30 kHz-800 MHz), PFG-NMR, and MD simulations to constrain models; and (3) thoughtful application of Nordio's (anisotropic rotation) and Torrey's (diffusion) theories.

While the study is fundamentally sound and novel, minor revisions would strengthen it further:

(a) explicit discussion of error propagation in CSA fitting and model limitations (e.g., transient ion pairing effects on diffusion).

R: We thank the reviewer for this important observation. The uncertainties associated with the CSA values (in ppm) are reported in Table 2. A comment on this regarding was added to Page *11* *(These uncertainties were obtained considering the uncertainty of the experimental relaxation rates and fitting residues for a χ^2 normalized to 1).* Within these error margins, no significant deviations in the relaxation profiles are observed, supporting the robustness of the CSA fitting.

Regarding model limitations and the potential influence of transient ion pairing on diffusion, we note that such effects would primarily affect the low-frequency region, where diffusion-driven mechanisms dominate, as reported by Danuta Kruk et al. (Int. J. Mol. Sci. **2022**, 23, 1688). However, the good agreement between the experimental data and the fitted curves across the full frequency range suggests that these transient effects do not significantly affect the accuracy of the extracted parameters under the conditions studied, considering that the measured self-diffusion coefficients used as a fixed parameter already average the translational motion of the ions. A comment was added on Page 10: "*Note that the use of the measured self-diffusion coefficients D for cations (^{1}H domain) and anions (^{19}F domain) in the relaxation model automatically includes eventual ion association and correlated motions.*" Additional comment addressing this point has been also included in the main manuscript (Page 16): "*The good agreement between the experimental data and the fitting curves across the full frequency range serves as a self-consistency proof of the reliability of the relaxation*

model used to describe the observed R_1 profiles. Consequently, any attempt to deconvolute model deviations due to the presence of ion pairs would remain inconclusive."

(b) improved data presentation through annotated figures (e.g., labeled molecular structures) and inclusion of raw NMRD data in SI (add raw data in SI).

R: We thank the reviewer for this helpful suggestion. In response, we have added the full names of the molecular structures in Figure 1 to improve clarity. Additionally, the raw NMRD data have been included in the Supporting Information (Table S4-S5).

(c) deeper contextualization of CSA values versus literature and broader implications for IL design.

R: We thank the reviewer for this insightful suggestion. To the best of our knowledge, there are no reported ^{19}F CSA values for fluorine in ionic liquids. However, a careful review of the literature reveals that ^{19}F CSA values extracted from MAS spectra of polycrystalline amino acids using Herzfeld–Berger analysis ranges from approximately 10 to 75 ppm (Ref. 47-48 in the main manuscript), which is consistent with the values reported in our study. Additionally, the CSA pattern of the fluorine nucleus in the FSI$^-$ anion of an organic ionic plastic crystal was recorded by solid-state NMR and discussed by Liu Jin et al. (J. Phys. Chem. B, 2017; Ref. 49 in the main manuscript). Although this investigation was not conducted in the context of ionic liquids, it provides further support for the CSA values reported here and underscores the novelty of our findings in the relaxation dynamics.

This has been included in the manuscript as follows on page 14: *"The C_{SA} values reported here (4–69 ppm) are consistent with those found in the literature for polycrystalline amino acids (10–75 ppm)[40,41], confirming that they are physically meaningful. Additionally, CSA patterns have been experimentally observed for the FSI$^-$ anion in organic ionic plastic crystal materials by solid-state NMR[42], further supporting its role as an efficient ^{19}F relaxation mechanism, as described here."*

In terms of broader implications, we have also included the following sentence in the revised manuscript, as suggested by Reviewer 2 (page 15): *"Together, these insights provide new guidelines for interpreting ^{19}F NMR data in fluorine-containing ionic systems and highlight the potential of CSA-sensitive nuclei in the rational design of ionic liquid-based materials."*

Reviewer: 2

Comments:
This article presents careful measurements and a theoretically driven fitting approach to assessing the different spin magnetization/motional effects that influence T1 relaxation in some key representative ionic liquids (ILs). Using NMR T1 relaxation dispersion is a key experimental method for understanding how ILs translate and rotate. This dynamical information helps drive the uses of ILs in many applications including battery electrolytes and carbon capture. The key insight here is that the chemical shift anisotropy (CSA) for 19F has a strong effect on the 19F relaxation rates, and that this CSA effect provides new and systematic information about the rotations and translations of fluorinated anions. The authors wisely study the common series BF4-, PF6-, TFSI-, and FSI- and find very useful motional insights. This article is written clearly and should be published in JPC Letters. Below are a number of points that the authors should address before publication.

1) On the bottom of p. 2 in the sentence that ends with ref 6, I suggest using "T1" in the definition of NMRD and R1. For uninitiated readers, this might make them more enticed to read as many more physical chemists have heard of T1 than R1.

R: We thank the reviewer for this insightful suggestion. To improve clarity for a broader audience, we have revised the sentence in the main manuscript to include both terms. The new version reads (Page 2): *"…by measuring the spin-lattice relaxation rate (R_1), which is the reciprocal of the spin-lattice relaxation time T_1…"*

2) In the middle of p. 3, you state that the BPP and Cole Davidson models "…considered in the context of small and symmetric molecules, it relies on the assumption of an isotropic reorientation and a single averaged correlation time." At least for BPP, this also relies an another huge assumption - that the coupled spins are covalently bonded to the same molecule, and/or have a well-defined distance between specific pairs of nuclei during the timescale of the experiment. This distance is definitely never well defined for ILs, and indeed the idea that NMR T1 or NOE can report on distances has been challenged based on distant dipolar field effects. There is a paper by H. Weingartner on this around 2010-2014, but I cannot find it at the moment. It is also well known, although rarely spoken, that distances obtained from T1 relaxivities on MRI contrast agents are only for relative comparison purposes since the BPP theory is violated (no proximity of a given water to a given agent molecule during the T1 measurement) for every case of water coordinating with a contrast agent. I encourage you to rewrite some of your statements about distances in the paper according to these concepts. Heteronuclear T1 effects necessarily involve only intermolecular terms in the case of these ILs, so the information from these T1 terms is particularly suspect.

R: We thank the reviewer for this important comment. We fully agree with the concerns raised and, for this reason, we employed the Nordio model to better describe the anisotropic molecular reorientation dynamics of elongated ions. In this model, the molecular director is defined from the

molecular coordinates, as detailed in *Field-Cycling NMR Relaxometry: Instrumentation, Model Theories, and Applications* (2019), Chapter 11. We have added a comment on Page 8 of the main manuscript referring to that: *"This approach is particularly useful for describing the rotational reorientation of elongated and asymmetrical molecules, which lack well-defined intramolecular distances between specific spin pairs."*

For the small and symmetric anions PF_6^- and BF_4^-, we used the BPP approach, with the intramolecular distance r_{FF} estimated independently from molecular dynamics (MD) simulations (Figure S3 and Table S3). We acknowledge that these distances cannot be considered as constant. Indeed, they were used as fixed values in the fitting because they were obtained by other experimental/computational techniques; and as a best approximation, we adopted values within the most intense peak in the distance distribution as the representative r_{FF} value used in the BPP-based analysis (Figure S2). A similar procedure was followed to determine the intermolecular distances d_{ii} and *dij*, also obtained from MD simulations (Figure S3).

We have revised the manuscript to better reflect these model assumptions and limitations and included the following clarification (Page 9-10):

"When calculating relaxation contributions associated with translational diffusion, only intermolecular interactions are relevant. For intermolecular homonuclear contributions, cation–cation and anion–anion distances were considered to determine the average intermolecular spin distance d_{ii}. For heteronuclear contributions, only cation–anion (counter-ion) distances (d_{ij}) were used, as ^{19}F nuclei are located exclusively in the anion and ^{1}H nuclei in the cation. All distance values were obtained from molecular dynamics simulations, as described in the Supporting Information."

We also clarified the content of Table 1 (label adjusted as: *Table 1: ^{1}H and ^{19}F model parameters used as fixed values in the model fitting procedures obtained: (a) experimentally or (b) by MD simulations, with representative values corresponding to the distance from the most intense peak in the RDF distribution, as described in the SI (Figure S2 and S3)…)*. and revised the intramolecular distance r_{FF} for PF_6^-, $BF_4^-$and FSI^- to reflect the value of the most intense peak in the MD-derived distribution, as reported in Fig. S2-S3. The plot on Fig 4 and values on Table 2 were also revised accordingly.

3) I applaud your use of the anisotropic rotation theory (originally from liquid crystal NMR) to assess relaxation contributions for FSI and TFSI. These are anisotropic molecules, even though their D and CSA anisotropy terms are averaged out in the spectra (well stated!). This aspect seems to be novel and exciting, and could perhaps be emphasized more in the abstract or intro? Also, you may be interested to look at a ref involving anisotropic spin interactions in FSI- in an OIPC (https://doi.org/10.1021/acs.jpcb.7b02780, authors L. Jin… J. Pringle, JPCB, 2017). In the OIPC, the CSA terms/patterns do not average out and do appear in the spectra!

R: We thank the reviewer for the insightful comment and for highlighting the literature on CSA in organic ionic plastic crystals. We have now included this reference in our citations (Page 15): *The C_{SA} values reported here (4–69 ppm) are consistent with those found in the literature for polycrystalline amino acids (10–75 ppm)[47,48], confirming that they are physically meaningful. Additionally, CSA patterns have been experimentally observed for the FSI^- anion in organic ionic plastic crystal materials by solid-state NMR[49], further supporting its role as an efficient ^{19}F relaxation mechanism, as described here.*

We have also emphasized the key point suggested by the reviewer by expanding the discussion on Page 15 to highlight the novelty of our findings. Specifically, we added:

"This relaxation mechanism has often been overlooked. Although CSA effects are averaged out in the spectra of isotropic liquids due to fast molecular motion, the time-dependent fluctuations of the CSA tensor remain a significant and efficient relaxation pathway—particularly for anisotropic molecules at high magnetic fields."

4) In a few places where named models are mentioned (BPP, Cole Davidson, Nordio), key original references are left out, or only included much later in the paper. Please include the basic/original refs when the models/names are first mentioned.

R: We have inserted key references as suggested by the reviewer:

(13) Silva, G. M. C.; Beira, M. J.; Morgado, P.; Branco, L. C.; Sebastião, P. J.; Canongia Lopes, J. N.; Filipe, E. J. M. Ionic Liquids with Hydrogenated and Perfluorinated Chains: Structural Study of the [P6,6,6,14][FnCOO] n = 7, 9, 11. Checking the Existence of Polar – Hydrogenated – Perfluorinated Triphilic Continuity. J Mol Liq 2022, 367, 120506. https://doi.org/10.1016/j.molliq.2022.120506.

(14)Kruk, D.; Masiewicz, E.; Lotarska, S.; Markiewicz, R.; Jurga, S. Correlated Dynamics in Ionic Liquids by Means of NMR Relaxometry: Butyltriethylammonium Bis(Trifluoromethanesulfonyl)Imide as an Example. Int J Mol Sci 2021, 22 (17), 9117. https://doi.org/10.3390/ijms22179117.

(15)Honegger, P.; Overbeck, V.; Strate, A.; Appelhagen, A.; Sappl, M.; Heid, E.; Schröder, C.; Ludwig, R.; Steinhauser, O. Understanding the Nature of Nuclear Magnetic Resonance Relaxation by Means of Fast-Field-Cycling Relaxometry and Molecular Dynamics Simulations—The Validity of Relaxation Models. J Phys Chem Lett 2020, 11 (6), 2165–2170. https://doi.org/10.1021/acs.jpclett.0c00087.

(16)Ordikhani Seyedlar, A.; Stapf, S.; Mattea, C. Cation Dynamics in Supercooled and Solid Alkyl Methylimidazolium Bromide Ionic Liquids. J Phys Chem B 2017, 121 (21), 5363–5373. https://doi.org/10.1021/acs.jpcb.7b01712.

(17)Kruk, D.; Meier, R.; Rachocki, A.; Korpała, A.; Singh, R. K.; Rössler, E. A. Determining Diffusion Coefficients of Ionic Liquids by Means of Field Cycling Nuclear Magnetic Resonance Relaxometry. J Chem Phys 2014, 140 (24). https://doi.org/10.1063/1.4882064.

(18)Becher, M.; Steinrücken, E.; Vogel, M. On the Relation between Reorientation and Diffusion in Glass-Forming Ionic Liquids with Micro-Heterogeneous Structures. J Chem Phys 2019, 151 (19). https://doi.org/10.1063/1.5128420.

(19)Wencka, M.; Apih, T.; Korošec, R. C.; Jenczyk, J.; Jarek, M.; Szutkowski, K.; Jurga, S.; Dolinšek, J. Molecular Dynamics of 1-Ethyl-3-Methylimidazolium Triflate Ionic Liquid Studied by 1 H and 19 F Nuclear Magnetic Resonances. Physical Chemistry Chemical Physics 2017, 19 (23), 15368–15376. https://doi.org/10.1039/C7CP01045A.

5) I have a personal problem with the idea of a jump distance in liquid systems (with usually continuously varying jump distances), but you have written/applied the relevant model in as appropriate form as possible. I find it encouraging that the jump distances you extract from the R1 fits are pretty close to the anion-anion distances and other relevant average lengths in these systems. This relatively close matching usually does not come out of similar model fits when finding tau_D using dielectric spectroscopy or dielectric combined with NMR diffusion coeffs.

R: *We thank the reviewer for his/her appreciation of this work.*

6) The derivations on p. 9 (eqs. 7-11) include some text that does not transition well to the next equation. Please consider rewriting this section to add more steps or more clearly delineate which equations go with which others. For example, when going from eq. 10 to 11, I originally thought you were determining 11 from 10, but it looks like you are simply introducing 11 from a reference and it is independent of 10.

R: *Before introducing Eq. 11, we have included the following sentence to avoid misinterpretation (Page 10): "In addition to the dipolar relaxation mechanisms described above, fluctuations in the local magnetic field due to chemical shift anisotropy (CSA) can also contribute to spin relaxation, particularly for ^{19}F nuclei."*

7) Reference 16 appears to be incomplete.

R: We have completed the details of Ref. 16 (now Ref. 23 in the revised version).

(23) Torrey, H. C. Nuclear Spin Relaxation by Translational Diffusion. Physical Review **1953**, 92 (4). https://doi.org/10.1103/PhysRev.92.962.*

8) Suggested rewriting of last sentence of main paper: "…and highlight the potential to use information from CSA-sensitive nuclei to bolster the rational design of ionic liquid-based materials."

R: We thank the reviewer, and we have rewritten the last sentence of the paper to (Page 16):

"Together, these insights provide new guidelines for interpreting ^{19}F NMR data in fluorine-containing ionic and highlight the potential of CSA-sensitive nuclei in the rational design of ionic liquid-based materials."
